# Research on the Differences in Phenotypic Traits and Nutritional Composition of Acer Truncatum Bunge Seeds from Various Regions

**DOI:** 10.3390/foods12132444

**Published:** 2023-06-21

**Authors:** Xiaona Le, Wen Zhang, Guotao Sun, Jinshuan Fan, Mingqiang Zhu

**Affiliations:** 1 College of Mechanical and Electronic Engineering, Northwest A&F University, Yangling 712100, China; lxn@nwafu.edu.cn (X.L.); guotao_sun@nwsuaf.edu.cn (G.S.); 2 Northwest Research Center of Rural Renewable Energy Exploitation and Utilization of M.O.A, Northwest A&F University, Yangling 712100, China; wen30511011@163.com; 3 College of Forestry, Northwest A&F University, Yangling 712100, China; fanjinshuan@163.com

**Keywords:** Acer truncatum Bunge, edible oil resources, fatty acids, organic compounds, selection of the optimal region

## Abstract

Acer truncatum Bunge (ATB) is an excellent edible woody oil tree species since it bears a huge amount of fruit and has strong adaptability to be widely cultivated. Selecting an optimal cultivation region for ATB is crucial to improving China’s woody oil industrialization. Chemical analysis, correlation analysis, and affiliation function values were used in the present research to systematically analyze the phenotypic traits, organic compound content, and seed oil chemical composition of the seeds of ATB from nine regions. The average contents of oil, protein, and soluble sugar in ATB seeds were 43.30%, 17.40%, and 4.57%, respectively. Thirteen fatty acids were identified from ATB seed oil, the highest content of which was linoleic acid (37.95%) and nervonic acid content was 5–7%. The maximum content of unsaturated fatty acids in ATB seed oil was 90.09%. Alpha-tocopherol content was up to 80.75 mg/100 g. The degree of variation in seed quality traits (25.96%) was stronger than in morphological traits (14.55%). Compared to environmental factors, the phenotypic traits of seeds contribute more to organic compounds and fatty acids. Combining the values of the indicator affiliation functions, Gilgarang, Tongliao, Inner Mongolia was selected as the optimal source of ATB for fruit applications from nine regions.

## 1. Introduction

As a populous country, China has been suffering from a low self-sufficiency rate (31%) in edible vegetable oils. In the last decade, China has been overdependent on external edible oil resources, relying on permanent imports with a pronounced growth trend in consumption [1]. The domestic edible oil market is heavily dominated by foreign markets, for example, the palm oil consumed today is totally dependent on imports [2]. Developing the wood-based oilseed industry can effectively increase Chinese oil resources and enhance the self-sufficiency of edible oils. At the same time, international attention is switched to developing wood-based edible oils to mitigate the shortage of edible oils [1]. The most common woody oil seeds have not been extensively developed due to the limitations of production area (tea seed oil, palm oil, coconut oil, and olive oil) and economic benefits (walnut oil). Recently, researchers have gradually shifted their attention to Acer truncatum Bunge (ATB) [3]. Meanwhile, the ATB Engineering Technology Research Center was established in 2018.

**Figure 1:**
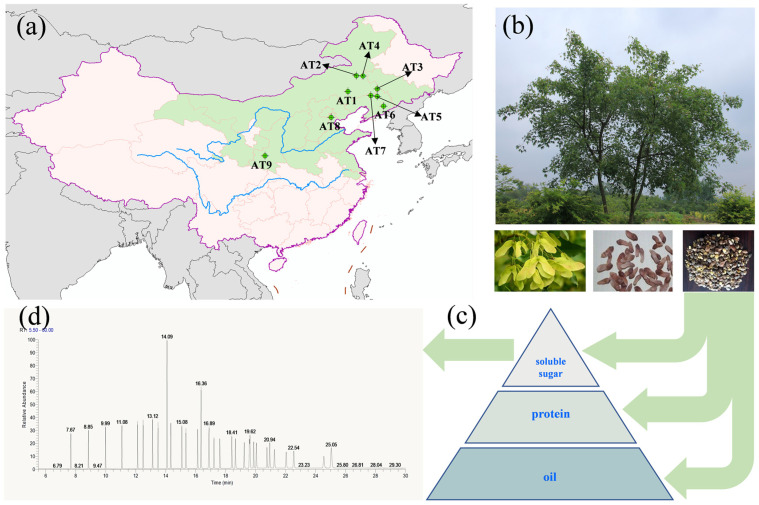
Collection and treatment of Acer truncatum Bunge seeds: (**a**) location of Acer truncatum Bunge seeds collected from different origins, (**b**) the morphology of the Acer truncatum Bunge tree, fruit, and seeds, (**c**) the composition of organic compounds in Acer truncatum Bunge seeds, (**d**) the chromatogram of standard mixtures (fatty acid methyl esters MIX, C4-C24).

As a particular truncatum and lofty deciduous leaf arbor in China, ATB is grown in several provinces (Jilin, Liaoning, Inner Mongolia, Hebei, Shanxi, Shandong, north of Jiangsu, Henan, Shaanxi, and Gansu) and is widely cultivated for its adaptability and fruit-bearing capacity (Figure 1a) [4]. ATB seeds have a particularly favorable oil content and the oil is abundant in unsaturated fatty acids (UFAs, 90%) with valuable linoleic acid (C18:2), linolenic acid (C18:3), and nervonic acid (C24:1) [5,6]. Recently, the oil extracted from the seed kernel of A. truncatum has been listed as a new resource of edible oil by the National Health Commission of the People's Republic of China [7]. Additionally, ATB is an excellent species of woody oilseed that does not compete with cereal for land distribution. ATB can also be closely integrated with ecological construction to better carry out work on ecological environment construction, poverty alleviation in mountainous areas, and expansion of new industries [8].

Research showed that ATB seed oil has a wealth of C24:1 and UFAs. C24:1 is an extremely vital nutrient for the brain and brain nerves. Inadequate intake of C24:1 will cause loss of memory and intellect, which affects the intellectual progress of infants and children and enhances the dementia risk of Alzheimer’s disease [9,10,11]. UFAs with long chains are molecules that preserve the membrane's relative fluidity for the proper physiological function of cells [12]. Representative monounsaturated fatty acids (MUFAs, with only one unsaturated bond in the carbon chain) include oleic acid (C18:1), while polyunsaturated fatty acids (PUFAs, i.e., two or more unsaturated bonds in the carbon chain) include C18:2, C18:3, and arachidonic acid [12]. PUFA is essential in human physiology and has a positive function in blood vessels, immunity, cells, and cancer. Clinical research has shown that PUFA intake can reduce undesirable lipids such as cholesterol in the blood. In addition, γ-C18:2 can significantly suppress the content of triglycerides, cholesterol, and other lipids (more than 60% lipid) and prevents the formation of blood thrombi [12]. The majority of the essential UFAs, such as C18:2 and C18:3, cannot be synthesized by humans and have to be obtained from external sources. Previous investigations have reported that plants (avocados, nuts, and olives) are rich in UFA, whereas these industries have not been fully exploited in accordance with their content and availability [13,14,15]. In summary, ATB has great potential for development. However, the distribution of ATB in China straddles a wide range and the climate, ecology, latitude, and longitude of each region are quite heterogeneous [5,6]. The consequences of regional variations on the phenotype and nutritional composition of ATB seeds are not yet clarified. Therefore, it is necessary to investigate the optimal cultivation region for ATB in order to promote the development of the woody oilseed industry in China.

In this preliminary investigation, nine representative ATB regions were selected through a data search and field inspection. The phenotypic properties (open angle, long diameter, transverse diameter, longitudinal diameter, growth mark length, weight per hundred kernels, and kernel yield) of ATB seeds and the variation of characteristics of nutritional composition (oil, protein, soluble sugar, fatty acids) of the nine regions were systematically investigated by correlation analysis. In addition, the optimal ATB region for fruits was identified by affiliation function. Finally, the program is intended to provide data and theoretical foundations for the selection and breeding of the best ATB species and to promote the development of ATB resources to alleviate the tension of oil consumption in China.

## 2. Materials and Methods

### 2.1. Plant Materials

One hundred samaras of ATB grown naturally were collected from nine natural core distribution areas of ATB in Liaoning, Inner Mongolia, Shandong, and Shaanxi Provinces (Table 1 and Figure 1a). The fruit was collected following the principle of random sampling. Approximately 10 individual disease-free, insect-pest-free plants (20 years of age or older) were selected from each sample collection area. The latitude, longitude, and elevation of the above areas were observed with the BeiDou Navigation Satellite System, and meteorological factors were found by consulting local meteorological services (Table 1).

### 2.2. Phenotypic Traits of Samaras and Seeds

Fifty undamaged samaras and seeds from each ATB accession were randomly selected for morphological analysis (Figure 1b). The open angle, long diameter, transverse diameter, longitudinal diameter, growth mark length, weight per hundred kernels, and kernel yield of samaras were measured (Table S1). The long diameter, transverse diameter, longitudinal diameter, weight per hundred kernels, and kernel yield rate of seeds were measured (Table S2). 

### 2.3. Determination of Oil, Protein, and Soluble Sugar Content in ATB Seeds

#### 2.3.1. Extension of Seed Oil and Determination of Its Content

The content of oil in ATB seeds was determined by using the Soxhlet extraction method. Ten grams of powdered ATB seeds was accurately weighed, sieved (20–40 mesh), wrapped in a filter paper cartridge, and then put into a Soxhlet extractor. Diethyl ether was used as the solvent, and reflux extraction was performed at 50 °C for 6 h. After the oil was completely leached out, the diethyl ether was recovered to obtain a pale yellow liquid, which was rotary evaporated at 50 °C for 30 min and placed in an oven at 100 °C for constant temperature drying to obtain ATB oil. The residues extracted were subjected to further analysis.

#### 2.3.2. Protein

First, 0.1 g of degreasing powdered (residues in Section 2.3.1) ATB seeds was accurately weighed and transferred into a dry digestion tube. Two grams of catalyst (copper sulfate pentahydrate: potassium sulfate = 1:9) and sulfuric acid (5 mL) were added to the tube. The tube was shaken well and placed into a digester which has been preheated to 420 °C and reacted for 1 h. The digestion tubes were removed and cooled. The Kjeldahl method was adopted to detect the content of total protein in ATB seeds [16].

#### 2.3.3. Soluble Sugar

The content of soluble sugar in ATB seeds was established by the anthrone sulfate method. Different concentrations (0, 0.2.0.4, 0.6, 0.8, 1.0 mg/mL) of glucose solution were added to the stoppered test tube, anthraquinone sulfate (5 mL) was added to the test tube, and the mixture was shaken well. After opening the stopper the tube was placed in a boiling water bath for 10 min. After the reactants were cooled to room temperature, the absorbance values were measured at 625 nm. The standard curve was plotted using the content of glucose as the horizontal coordinate and the absorbance value as the vertical coordinate. The standard curve was obtained as follows: y = 2.39x + 0.2314 (R^2^ = 0.9911).

Then, 0.1g of degreasing powdered (residues in Section 2.3.1) ATB seeds was put into a test tube, distilled water (10 mL) was added to the test tube, and the tube was heated in a boiling water bath for 10 min. After centrifugation (8000 r/min) for 1 h, the supernatant was fixed at 25 mL in a volumetric flask. Soluble sugar extract (0.5 mL) was pipetted into a test tube, anthraquinone sulfate (5 mL) was added, and the tube was boiled in a water bath for 10 min. After the reaction solution had cooled, the absorbance value was rapidly measured at 625 nm. The content of soluble sugar in the ATB seeds was calculated from the absorbance values of the sample solutions according to the standard curve of glucose content.

### 2.4. Determination of the Nutritional Composition of oil in ATB Seeds 

#### 2.4.1. Determination of Alpha-Tocopherol Content

The seed oil of ATB (0.1 mL) was precisely measured in a test tube, anhydrous ethanol (4 mL) was added to the tube, and then the mixture was extracted with the aid of ultrasound for 0.5 h. After extraction, the extracts were centrifuged (4000 r/min) for 10 min and then retained in the supernatant. The supernatant was filtered by a 0.45 μm filter and the content of alpha-tocopherol was determined by HPLC. Five microliters of supernatant was passed through a C18 column (250 mm × 4.6 mm, 5 μm) at a flow rate of 1 mL/min with methanol (100%) as the eluent. The eluent from the column was passed through a UV detector. Separations were performed at 25 °C, and the detection of UV elution profiles was at 280 nm. The content of alpha-tocopherol was calculated multiple times by the standard curves based on the responding peak areas in HPLC.

#### 2.4.2. Composition Analysis of Fatty Acid by Methyl Esterification

The methyl esterification of ATB oil was carried out by alkali catalysis. ATB oil (0.1 mL) was added to the test tube, n-hepatane (2 mL) was added to dissolve the ATB oil, and 0.5% KOH/CH_3_OH (2 mL) was added to the test tube. The mixture was shaken to make the solution fully react. The reaction was carried out in a water bath at 80 °C for 5–10 min, and the supernatant was taken and subjected to on-board determination. The chemical composition of ATB oil was analyzed with a GC-MS equipped with a quadrupole MS detector, split/splitless injector, autosampler (Thermo Fisher, USA), and an elastic quartz DB-FFAP MS capillary GC column (30 m × 0.25 mm, 0.25 μm). The oven temperature was controlled as follows: held at 70 °C for 1 min, ramped at 10 °C/min to 200 °C, ramped at 5 °C/min to 230 °C, and held for 15 min. The nitrogen carrier gas was maintained at a constant flow rate of 0.4 mL/min. Linear velocity mode was used and set at the rate of 40 cm/s. The injector temperature was set at 230 °C with a split injection ratio of 70:1, and the detector temperature was 230 °C. Electron-impact ionization (EI) mode was used and set to 70 eV. The ion source temperature was 240 °C. The mass scan range was 40–600 amu, retention time was 10 min, and scan mode was full scan. The injection volume was 1 μL, and each sample was injected in triplicate (Figure 1d).

### 2.5. Selection of Optimal Sources of ATB

The method of affiliation function in fuzzy mathematics was used to screen for optimal ATB source sites. The affiliation values were obtained for the phenotypic traits and the content of the main nutritional ingredients of ATB species. The quality of the ATB of different regions increased with the growth of the average affiliation value. The affiliation value of each index is calculated as follows:

$$Affiliation\;function\;value=\frac{A-A_{min}}{\left(A_{max}-A_{min}\right)}\times 100\%$$

$$Inverse\;affiliation\;function\;value=1-\frac{A-A_{min}}{\left(A_{max}-A_{min}\right)}\times 100\%$$

where “*A*” is the measured value of an indicator for a region, “*A_max_*” is the maximum value of this indicator among the 9 regions, and “*A_min_*” is the minimum value of the indicator.

### 2.6. Statistical Analysis

SPSS 22.0 (SPSS, Michigan Avenue, Chicago, IL, USA) was used to carry out one-way ANOVA (Duncan’s method for significance of differences, *p* < 0.05 indicates a significant difference), correlation, and multiple regression. All data were presented as means with their standard error of the mean of 3 replicates.

## 3. Results

### 3.1. Morphological Diversity of ATB Fruits and Kernel Seeds

Phenotypic trait variation is the result of a stress response of the plant for adapting the different environmental conditions [17], which is of great importance in the adaptation and phylogeny of species. For fruit-producing economic tree species, the phenotypic traits of fruits and seeds are particularly important. The variable coefficient is usually used to indicate the degree of dispersion of traits, which shows a positive correlation between them. Therefore, the variable coefficient indirectly indicates richer phenotypic diversity.

The results of phenotypic traits of ATB fruits and seeds are shown in Tables S1 and S2. The analysis showed that ATB fruits and seeds’ phenotypic traits differed significantly (*p* < 0.05) between different regions. The phenotypic shapes of ATB fruits and seeds were evaluated according to morphology (long diameter, transverse diameter, longitudinal diameter, growth mark length, open angle) and qualitative indicators (weight per hundred kernels, kernel yield). In terms of morphological traits, the largest variable coefficient in ATB fruit morphology was longitudinal diameter (15.58%) and the smallest was the long diameter (11.35%). The largest variable coefficient in ATB seed morphology was the longitudinal diameter (25.46%) and the smallest was the long diameter (13.2%). The average variable coefficient of fruit morphology (12.83%) was smaller than that of the seed morphology (17.41%), therefore, the variation of seed morphology was richer than that of the fruit morphology in the nine regions of ATB. The variable coefficient of samara morphology is smaller than that of the seed morphology, which indicates that the environment has a broader influence on the phenotypic traits of seeds and is more abundant in diversity. This is consistent with the previous findings that phenotypic traits of natural Pinus sylvestris cones are relatively more robust than those of seeds [18] and that cone variation is weaker than seed variation amongst spruce populations [19]. In terms of qualitative traits, the variable coefficient was 33.34% (weight per hundred kernels) and 21.81% (kernel yield) for fruits and 36.12% (weight per hundred kernels) and 12.54% (kernel yield) for seeds. The average variable coefficient of quality traits reached 25.96%, while the average variable coefficient of morphological traits was only 14.55%. The ATB weight index is widely variable, while the external morphological index is rather steady, and this finding is consistent with the stability of the genetic characteristics of the morphological configuration of the seeds. This observation was also demonstrated by research on the natural variation of small-fruited oil tea seeds in Guizhou Province in China [20]. Based on the outcome of seed and fruit phenotypic measurements, it was found that individual seeds of ATB were larger in colder regions in northern China (AT1, AT3, AT4, AT5, AT6, AT7) than regions in southern China (AT8, AT9).

**Figure 2:**
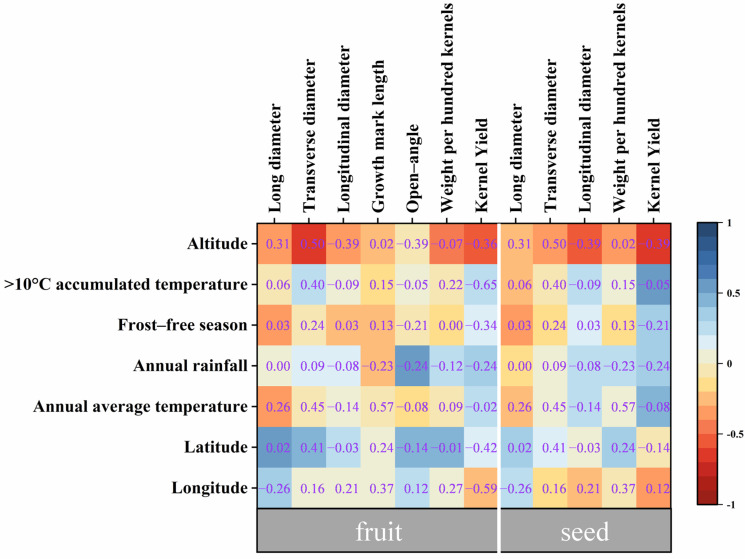
Influence of environmental factors on phenotypic traits of Acer truncatum Bunge fruits and seeds. Pearson correlation analysis was used.

The findings of correlation analysis between phenotypic traits of ATB and environmental factors (Figure 2) showed that seed kernel yield indicated a significant negative correlation with altitude (−0.7). In detail, the kernel yield of ATB seeds gradually decreased with the increase in altitude. Meanwhile, other traits were not significantly correlated with geographical environment factors. Overall, most of the environmental factors did not have a significant influence on ATB phenotypic traits, which implied that the phenotypic trait variation of ATB might be mainly due to gene regulation.

### 3.2. The Oil, Protein, and Soluble Sugar in ATB Seeds

**Figure 3:**
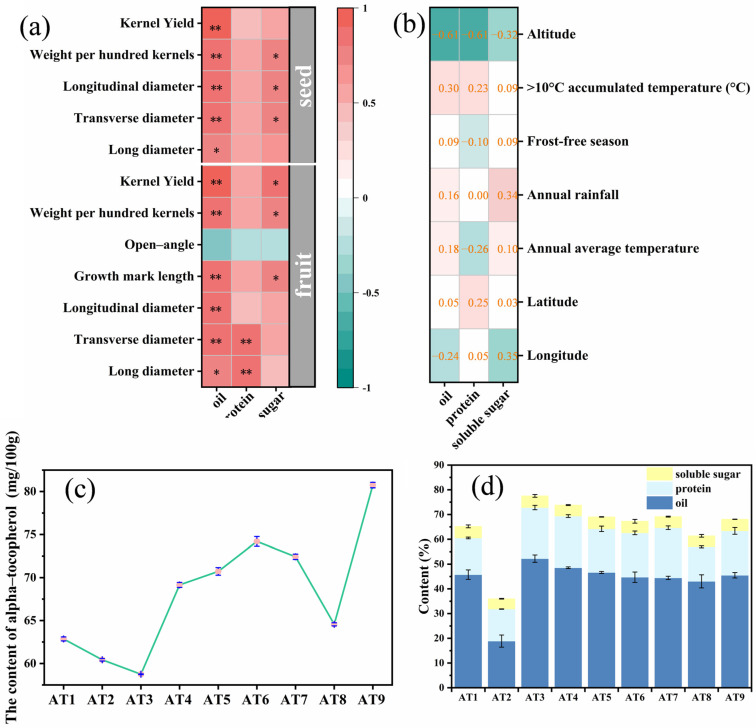
The Acer truncatum Bunge seeds’ influential factors and main component contents: (**a**) the influence of phenotypic traits on organic compounds in Acer truncatum Bunge seeds, (**b**) the influence of environmental factors on organic compounds in Acer truncatum Bunge seeds, (**c**) the content of alpha-tocopherol in Acer truncatum Bunge seed oil, (**d**) the content of oil, protein, and soluble sugars in Acer truncatum Bunge seed. Pearson correlation analysis was used, * *p* < 0.05, ** *p* < 0.01.

The seed contents and their intrinsic quality are related to the growth, development, and physiological activity of the plant, so the seed contents are an important indicator to identify the commerciality and usefulness of the fruits of woody edible oil trees [5]. ATB seeds contain a variety of chemical components and are an excellent-quality resource for oil extraction as well as protein extraction. The oil content of ATB seeds generally exceeds 40% and varies with the age of the tree, growth conditions, and other factors. The relative content of UFA in ATB oil is around 90%. The most important fatty acid components are C18:1, C18:2, and C24:1 [4,6]. The results of the correlation analysis between geographic environmental factors and ATB seed oil content (Figure 3b) showed that seed oil content had a significant negative correlation with altitude (−0.61), while none of the correlations with other geographic environmental factors were significant. Nevertheless, almost all ATB seed kernel phenotypic traits showed a significant positive correlation with oil content (Figure 3a). The highest seed oil content was in AT3 (52.23%) and the lowest was in AT2 (18.87%). In general, eight regions had seeds with oil content above 40% (Figure 3d). The variable coefficient of oil content of ATB seeds from different regions was 22.5% (Table 2), and their oil contents in the sequence of highest to lowest were AT3 (52.23%), AT4 (48.55%), AT5 (46.64%), AT1 (45.75%), AT9 (45.49%), AT6 (44.71%), AT7 (44.45%), AT8 (43.04%), AT2 (18.87%). The oil content of AT2 seeds was only one-third of that of AT3, which was probably because the geographical differences led to the poor fullness of the seeds in this region and the whitish seed kernels. From the index of oil content, AT3 and AT4 have great potential for oil utilization. Additionally, protein is one of the extremely valuable nutrients in ATB seeds.

Proteins are indispensable for life activities and play an extremely significant role in regulating physiological functions and maintaining metabolism. All essential parts of the human body structure require the participation of proteins [21]. ATB seeds contain more than 20% protein and have high nutritional value. Yang et al. [22] have demonstrated that ATB seed protein contains eight essential amino acids, including methionine, lysine, and valine. ATB seed protein has advantages over the common batan apricot kernels, hazelnut kernels, and walnut kernels, and is an ideal edible protein resource. Geographical environmental factors were not significantly correlated with the protein content of ATB seeds (Figure 3b). Furthermore, no ATB seeds kernel phenotypes except transverse diameter and long diameter were significantly correlated with protein content (Figure 3a). The variation coefficient of protein content in ATB seeds of different regions was 16.85% (Table 2). The largest content of protein in ATB seeds was that of AT4 (20.80%) and the smallest was that of AT2 (12.97%). From the index of protein content, AT3, AT4, and AT7 have a large potential for protein applications.

Soluble sugar is an essential class in the life cycle of plants. It plays an important role in plant hormone regulation, cold resistance, and drought resistance. Therefore, it is one of the indicators for evaluating the tolerance ability of plants [23,24]. Compared with oil or protein, the soluble sugar content in ATB seed kernels was relatively minor and can be exploited at a lower value (Figure 3d). The variation of soluble sugar content in ATB seed kernels from different regions was not significant since the variable coefficient was only 4.52 (Table 2). The largest content of soluble sugar in ATB seeds was that of AT5 (4.81%) and the smallest was that of AT2 (4.16%). In brief, the nutrient composition of ATBs and phenotypic traits presented a relationship that suggested they are independent and potentially associated with each other. Hence, comprehensive analysis and selection can be undertaken according to specific requirements in order to achieve efficient selections.

### 3.3. The Composition of Fatty Acid in ATB Seed Oil

The percentages of individual fatty acids present in the oil of ATB seeds are presented in Table 3. The one-way ANOVA investigated the effect of provenance on the fatty acid composition and showed significant differences in the content of major fatty acids between provenances, confirming the biodiversity of the ATB germplasm. The main fatty acids existing in ATB seed oil were linoleic acid (C18:2, 29.66–37.95%) and oleic acid (C18:1, 22.38–26.79%), followed by erucic acid (C22:1, 14.15–17.32%), cis-11-eicosanoic acid (C20:1, 7.40–8.68%), nervonic acid (C24:1, 3.85–6.55%), palmitic acid (C16:0, 4.50–4.14%), stearic acid (C18:0, 1.79–2.76%), and linolenic acid (C18:3, 1.02–2.53%), and small amounts of behenic acid (C22:0, 0.59–0.95%), arachidic acid (C20:0, 0.22–0.30%), xylic acid (C24:0, 0.12–0.27%), hexadecenoic acid (C16:1, 0–0.12%) and heptadecanoic acid (C17:0, 0.07–0.12%). The contents of saturated fatty acids (SFAs) are extremely low, dominated by palmitic acid and C18:0, both with relative contents of below 10%. The contents of UFA are particularly high with slight variations around 90%, among which the relative contents of C18:1 and C18:2 sum to more than 50%, with a relatively minor proportion of C18:3 (1.0% to 2.53%). In this experiment, a total of 13 fatty acids were detected in the seed oil of ATB from different producing areas. Chang et al. [25] detected a total of 14 fatty acids in a study of the composition of ATB oils from 11 different production areas. Compared to the present research, cis-11,14-eicosadienoic acid was detected more and other components were similar. Most importantly, as one of the most exploitable components of fatty acid species, the content of C24:1 was in the range of 5–7%, which was close to findings in a previous study [6].

Recently, research indicated that excessive intake of SFA increases the risk of cardiovascular disease, diabetes, and obesity [26,27,28]. However, a few other scientists disagree and believe that there is almost no relationship between these diseases and SFA intake [29]. Despite the fact that the relationship between SFA and the health of humans is not conclusive, there are a growing number of reports suggesting that replacing SFA with UFA in the daily diet is beneficial in reducing the risk of disease [30,31,32]. The SFA content of the ATB seed oil in this work was only around 8.40–9.21%, which is significantly lower than that of the common avocados (13.41–19.25%), olives (11.20–12.87%), or macadamia nuts (10.80–22.20%) [14,33,34]. ATB seed oil is enriched with MUFA (58.30%) and PUFA (38.97%), which are beneficial for health promotion. C18:1 in the human diet was demonstrated to reduce low-density lipoprotein levels in the blood, suppress tumorigenesis, ameliorate inflammatory diseases, and improve blood pressure [30,35]. C18:2 and C18:3 have an invaluable function in protecting the heart and brain and in preventing atherosclerosis [30]. In addition to modulating cardiac ion channels, C18:3 also protects blood vessel health by converting to ox-lipoprotein, reducing inflammation and improving blood pressure [36]. However, elevated amounts of C22:1 have been reported to cause myocardial lipid deposition and cardiac lesions [37]. C24:1 was closely associated with the development and preservation of the brain and the biosynthesis and improvement of nerve cells. It has an important positive influence on treating neurological disorders, promoting brain development, improving memory, and delaying brain aging [36,38,39,40].

**Figure 4:**
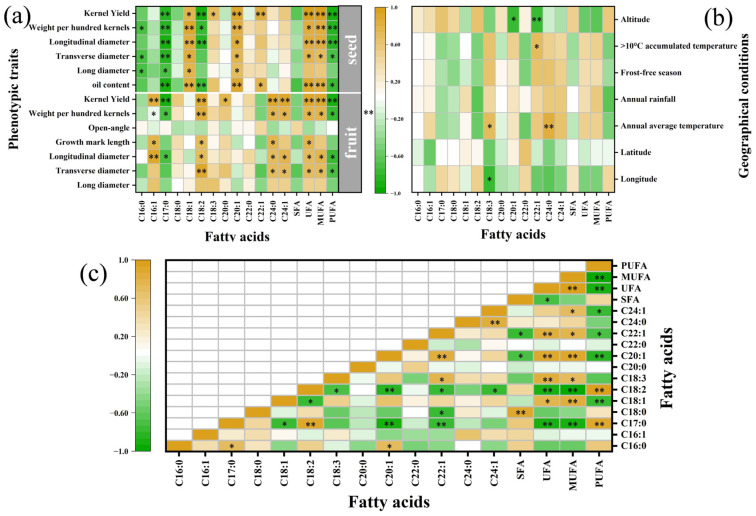
Influence factors of fatty acids in Acer truncatum Bunge seed oil: (**a**) influence of environmental factors on fatty acids, (**b**) influence of phenotypic traits on fatty acids, (**c**) influence of various fatty acids among each other. Pearson correlation analysis was used, * *p* < 0.05, ** *p* < 0.01.

Correlation analysis was carried out on the factors affecting the fatty acid content of ATB, shown in Figure 4, in which yellow represents a positive correlation and green a negative correlation (darker colors denote a stronger correlation). The variation in fatty acid fractions between regions was especially large and C16:1 showed the largest variable coefficient (55.56%) and C16:0 showed the smallest (4.17%) (Table 3). The largest variation coefficient of the fatty acid fractions was that of C16:1 (55.56%), followed by C17:0 (25%), C24:0 (25%), C18:3 (224.18%), C22:0 (18.18%), C24:1 (17.9%), C18:0 (12.81%), C20:0 (18.18%), C18:2 (7.28%), C22:1 (6.49%), C18:1 (5.21%), C20:1 (4.88%). The results of the correlation analysis between the contents of fatty acid fractions and environmental factors (Figure 4b) revealed a significant negative correlation between C18:3 and longitude (*p* < 0.05). In addition, there was a significant positive correlation with the annual average temperature (*p* < 0.05). C20:1 was significantly and negatively correlated with altitude (*p* < 0.05). C22:1 was significantly and positively correlated with >10 °C accumulated temperature (*p* < 0.05) and negatively correlated with altitude (*p* < 0.01). Lignocaine was significantly and positively correlated with annual average temperature (*p* < 0.05). C24:1 content was weakly negatively correlated with altitude, longitude, and latitude, while weakly positively correlated with annual rainfall, annual average temperature, and frost-free season. In addition to indicating a weak correlation between C24:1 content and environmental factors, the correlation trends of their environmental factors (altitude, annual rainfall) were also similar to those of [25]. The investigation results indicated that the content of C18:3 decreased significantly with the increase in longitude, while the content of C24:0 and C22:1 rose significantly with the increase in the annual average temperature. 

The correlation analysis of fatty acid fraction content with seed and fruit phenotypes (Figure 4a) revealed that the open angle had no significant influence on any fatty acid. The levels of both C18:0 and C22:0 were not significantly altered by any phenotype, differing in the situation where C20:0 exhibited a weak positive impact with all phenotypes. Meanwhile, C18:0 exhibited a weak positive association with the fruit phenotypes and a weak negative influence with the seed phenotypes. C18:2 was significantly and positively correlated with fruit phenotypes and negatively associated with seed phenotypes. C17:0 and PUFA were negatively related to fruit and seed in all phenotypes, and only a few traits were not significant (Figure 4a). UFA and MUFA were positively associated with all phenotypes of fruit and seeds (except open angle), and only a few traits were not significant (Figure 4a). C18:1, C18:2, and C24:1 are the most important nutritional indicators of ATB seed oil. Correlation analysis showed that as the oil content of the seeds increased, the C17:0 content decreased significantly. Moreover, when the C18:1, C18:2, C20:1, C22:1, UFA, MUFA, and PUFA contents rose significantly, C24:1 also improved (Figure 4a). Therefore, more desirable fatty acid ratios can be obtained by varying the seed oil content (regulating the content of UFA) and modifying the content of important fatty acid components (C18:1, C18:2, C18:3, and C24:1).

The main interrelationships between the identified fatty acids in ATB seed oil were determined by using Pearson’s correlation coefficient (Figure 4c). C18:2, one of the most abundant and essential UFAs in ATB seed oil, showed significant correlations with the majority of other fatty acids. The outcomes demonstrated that C18:2 had a positive correlation with all fatty acids except C16:0, C17:0, and C18:0, a strongly negative correlation with its precursor C18:1 (−0.80, *p* < 0.01), and a strongly positive correlation with C17:0 (0.90, *p* < 0.01). Although the amount of C24:1 was modest, it exerted an extremely strong physiological activity. C24:1 showed a significant positive correlation with C24:0 (0.84, *p* < 0.01) and a weak positive correlation with C22:1, while it showed a significant negative correlation with C18:2 (−0.76, *p* < 0.05). This is not entirely consistent with the results of Qiao et al. [6], and although both results showed that an increase in C24:1 content was accompanied by an increase in C22:1, Qiao et al. found a significant relationship between C24:1 and C22:1. Finally, despite C16:0 being the direct precursor of C18:0, no significant correlation was identified between these two fatty acids, and this was presumably because C18:0 was efficiently desaturated to C18:1 in ATB seed oil [14]. In addition, UFAs were found to be positively correlated with C18:1, C18:3, and C24:1, in contrast to a significant negative correlation with C18:2. Accordingly, a more desirable ratio of fatty acids can be obtained by changing the seed oil content to regulate the UFA content and modify the content of important fatty acid components (C18:1, C18:2, C18:3, and C24:1).

### 3.4. Alpha-Tocopherol of ATB Seed Oil

Alpha-tocopherol, as a fat-soluble vitamin with strong antioxidant properties, plays a significant role in antiaging, improving blood circulation, and enhancing the immunity of the body [41,42,43]. ATB seed oil is rich in alpha-tocopherol and has the potential to be exploited as an alpha-tocopherol resource. The results in Figure 3c showed that the average content of alpha-tocopherol in the nine regions of ATB seed oil production was 68.2 mg/100 g, which was higher than that of common palm oil, peanut oil, rapeseed oil, and other plant oils [44,45,46]. The highest content among the nine regions was that of AT9 (80.75 mg/100 g) and the lowest was that of AT3 (58.75 mg/100 g). The analysis showed significant variations (*p* < 0.05) in the alpha-tocopherol content of ATB seed oil between the nine provenances. The variable coefficient of alpha-tocopherol content in ATB seeds from the nine provenances was relatively minor (10.50%), indicating that the variation of alpha-tocopherol content was stabilized among the different provenances.

### 3.5. Screening for ATB Region

The selection of an excellent ATB region based on a single trait is subject to a high degree of uncertainty. Therefore, the quality and the content of the major nutritional components of the ATB seeds and fruits from nine regions were evaluated by single and multiple indexes to select the excellent ATB fruit regions. The mean subordinative function values of the different provenances were calculated based on the 10 phenotypic traits (including long diameter, transverse diameter, longitudinal diameter, weight per hundred kernels, kernel yield of fruits and seeds) associated with fruit and seed quality (Table S3). The poorest seed and fruit quality was found in AT2, which had a small seed size and low fullness and kernel yield, while AT7, AT3, and AT1 had superior seed and fruit quality. Eleven trait indicators (including long diameter, transverse diameter, longitudinal diameter, weight per hundred kernels, kernel yield of fruits and seeds) related to the protein content of ATB were chosen to calculate the mean subordinative function values for the nine regions (Table S4). AT3, AT4, and AT7 have a high potential for protein resource exploitation as a result of their higher protein content and excellent seed and fruit quality. The contents of oil, UFA, and C24:1 and the phenological traits (long diameter, transverse diameter, longitudinal diameter, weight per hundred kernels, kernel yield of fruits and seeds) were adopted as the reference for a comprehensive assessment of the oil quality of the nine regions given that these parameters were significantly and positively correlated with the oil content of ATB (Table S5). AT3 had a great potential for oil applications. The combination of the outcomes of the above indicators revealed that AT3 has the multiple advantages of excellent seed quality, plentiful oil resources, better oil quality, and higher protein content. Therefore, AT3 was selected as an excellent source of ATB and can be used for exploitation in ordinary foods, feed additives, cosmetics, functional foods, etc.

## 4. Conclusions

The phenotypic traits, nutritional composition content, and seed oil chemical composition of ATB seeds in different regions were examined by analysis of chemical, correlation, and affiliation function values. The results revealed that the average contents of oil, protein, and soluble sugar in the ATB seeds were 43.30%, 17.40%, and 4.57%, respectively. GC-MS was used to identify 13 fatty acids in the ATB seed oil, among which C18:2 had the highest content (37.95%), C24:1 content was in the range of 5–7%, and UFA content was up to 90.09%. The extent of variation in seed quality traits (25.96%) was higher than that in morphological traits (14.55%). Compared to environmental factors, the phenotypic traits of the seeds had a more significant effect on organic compounds and fatty acids. Finally, AT3 was preliminarily selected as the best fruit-producing ATB region among the nine regions evaluated considering the combined values of the affiliation functions of the indicators. The influence of harvest year on seed quality needs to be further investigated in coming research.

## Figures and Tables

**Table 1:** The geographical conditions of the sampling area.

Province	City	Locality	Label	Longitude (N)	Latitude (E)	Annual Average Temperature (°C)	Annual Rainfall (mm)	Frost-Free Season (d)	>10 °C Accumulated Temperature (°C)	Altitude (m)
Inner Mongolia	Chifeng	Wudan	AT1	43°13′50″	119°32′23″	7	375	115	1100	2025.0
	Chifeng	Bahrain Lindong	AT2	45°24′15″	120°58′52″	5	400	135	2000	1890.9
	Tongliao	Gilgarang	AT3	43°42′	123°42′	5.8	451.1	146	2900	308.4
	Hinggan League	Daichentala	AT4	45°18′28″	121°45′00″	5.6	388	120	3322	209.1
Liaoning	Shenyang	Faku	AT5	42°39′29″	123°45′14″	6.7	600	150	3219	106.0
	Fengcheng	Saima	AT6	41°06′	124°32′	8.1	1013.6	156	3210	500.0
	Fuxin	Zhangwu	AT7	42°51′	122°58′	7.1	510	156	2890	313.1
Shandong	Qufu		AT8	39°49′	117°13′	13.6	666.3	199	4457	60.5
Shaanxi	Yangling		AT9	34°20′	108°08′	12.9	635.1	211	4184	540.1

**Table 2:** The nutrition yield of Acer truncatum Bunge seeds from different locations (%, mean ± SD, *n* = 3).

Origin	Oil	Protein	Soluble Sugar
AT1	45.75 ± 1.93 ^b^	14.76 ± 0.34 ^d^	4.68 ± 0.57 ^c^
AT2	18.87 ± 2.46 ^c^	12.97 ± 0.09 ^d^	4.16 ± 0.15 ^f^
AT3	52.23 ± 1.48 ^a^	20.57 ± 0.88 ^a^	4.73 ± 0.57 ^b^
AT4	48.55 ± 0.35 ^ab^	20.80 ± 0.56 ^a^	4.46 ± 0.18 ^d^
AT5	46.64 ± 0.40 ^b^	17.57 ± 1.10 ^c^	4.81 ± 0.10 ^a^
AT6	44.71 ± 2.09 ^b^	17.86 ± 0.75 ^bc^	4.70 ± 0.77 ^c^
AT7	44.45 ± 0.66 ^b^	20.21 ± 0.73 ^ab^	4.48 ± 0.18 ^d^
AT8	43.04 ± 2.65 ^b^	13.92 ± 0.40 ^d^	4.41 ± 0.55 ^e^
AT9	45.49 ± 1.08 ^b^	17.90 ± 1.39 ^c^	4.69 ± 0.06 ^c^
CV (%)	22.05	16.85	4.52

Duncan’s multiple range test was used for analysis, and different lowercase letters in the same column indicate significant differences (*p* < 0.05, *n* = 3). CV: coefficient variation.

**Table 3:** Fatty acid composition and content in oil of Acer truncatum Bunge from different locations (%, mean ± SD).

	AT1	AT2	AT3	AT4	AT5	AT6	AT7	AT8	AT9	CV (%)
C16:0	4.71 ± 0.42 ^a^	5.14 ± 0.23 ^a^	4.55 ± 0.20 ^a^	4.80 ± 0.01 ^a^	5.02 ± 0.22 ^a^	4.83 ± 0.02 ^a^	4.50 ± 0.05 ^a^	4.86 ± 0.50 ^a^	4.83 ± 0.25 ^a^	4.17
C16:1	0.10 ± 0.25 ^a^	0.09 ± 0.15 ^a^	ND	0.10 ± 0.02 ^a^	ND	0.10 ± 0.02 ^a^	ND	0.10 ± 0.29 ^a^	0.12 ± 0.19 ^a^	55.56
C17:0	0.08 ± 0.29 ^a^	0.12 ± 0.16 ^a^	0.07 ± 0.07 ^a^	0.08 ± 0.02 ^a^	0.08 ± 0.15 ^a^	0.07 ± 0.02 ^a^	0.07 ± 0.05 ^a^	0.08 ± 0.34 ^a^	0.07 ± 0.21 ^a^	25.00
C18:0	2.59 ± 0.14 ^a^	2.74 ± 0.07 ^a^	1.79 ± 0.09 ^c^	2.76 ± 0.03 ^a^	2.32 ± 0.09 ^ab^	2.44 ± 0.02 ^ab^	2.65 ± 0.07 ^ab^	2.19 ± 0.23 ^b^	2.34 ± 0.04 ^ab^	12.81
C18:1	26.74 ± 0.21 ^ab^	22.38 ± 0.46 ^c^	25.28 ± 0.07 ^ab^	26.21 ± 1.24 ^a^	25.67 ± 0.08 ^ab^	26.79 ± 0.07 ^ab^	24.95 ± 0.14 ^ab^	25.09 ± 0.08 ^bc^	25.16 ± 0.45 ^ab^	5.21
C18:2	32.24 ± 1.61 ^bc^	37.95 ± 1.20 ^a^	32.12 ± 0.06 ^ab^	32.25 ± 0.77 ^abc^	32.65 ± 0.79 ^abc^	29.66 ± 0.26 ^b^	32.09 ± 0.01 ^abc^	30.26 ± 0.92 ^c^	31.27 ± 0.26 ^bc^	7.28
C18:3	1.76 ± 0.23 ^b^	1.02 ± 0.15 ^c^	1.92 ± 0.20 ^b^	1.75 ± 0.05 ^b^	2.01 ± 0.13 ^b^	1.90 ± 0.02 ^b^	1.35 ± 0.01 ^c^	2.16 ± 0.22 ^b^	2.53 ± 0.08 ^a^	24.18
C20:0	0.27 ± 0.40 ^a^	0.27 ± 0.24 ^a^	0.30 ± 0.20 ^a^	0.23 ± 0.08 ^a^	0.23 ± 0.24 ^a^	0.28 ± 0.03 ^a^	0.28 ± 0.06 ^a^	0.22 ± 0.43 ^a^	0.30 ± 0.29 ^a^	11.54
C20:1	8.24 ± 0.56 ^ab^	7.40 ± 0.25 ^b^	8.84 ± 0.40 ^a^	8.46 ± 0.21 ^a^	8.49 ± 0.22 ^a^	8.51 ± 0.04 ^a^	8.68 ± 0.13 ^a^	8.63 ± 0.67 ^a^	8.36 ± 0.76 ^a^	4.88
C22:0	0.81 ± 0.73 ^a^	0.77 ± 0.42 ^a^	0.90 ± 0.20 ^a^	0.72 ± 0.06 ^a^	0.67 ± 0.30 ^a^	0.95 ± 0.08 ^a^	0.93 ± 0.10 ^a^	0.59 ± 1.04 ^a^	0.61 ± 0.55 ^a^	18.18
C22:1	15.43 ± 1.24 ^ab^	14.15 ± 1.06 ^b^	17.24 ± 0.15 ^a^	15.66 ± 0.17 ^ab^	17.09 ± 0.79 ^a^	16.49 ± 0.02 ^a^	16.52 ± 0.35 ^a^	17.32 ± 1.82 ^a^	17.09 ± 2.07 ^a^	6.49
C24:0	0.19 ± 0.76 ^a^	0.17 ± 0.43 ^a^	0.12 ± 0.30 ^a^	0.19 ± 0.03 ^a^	0.15 ± 0.41 ^a^	0.24 ± 0.13 ^a^	0.22 ± 0.12 ^a^	0.27 ± 0.85 ^a^	0.25 ± 0.64 ^a^	25.00
C24:1	5.24 ± 0.39 ^abc^	4.06 ± 0.74 ^d^	4.76 ± 0.20 ^cd^	5.05 ± 0.02 ^bcd^	3.85 ± 0.29 ^e^	6.42 ± 0.02 ^ab^	5.77 ± 0.01 ^abc^	6.55 ± 0.34 ^a^	5.54 ± 0.07 ^abc^	17.9
SFA	8.65 ± 0.44 ^ab^	9.21 ± 0.38 ^a^	7.55 ± 0.35 ^ab^	8.78 ± 0.22 ^ab^	8.47 ± 0.31 ^ab^	8.81 ± 0.11 ^ab^	8.65 ± 0.13 ^ab^	8.21 ± 0.32 ^ab^	8.40 ± 0.52 ^ab^	5.39
UFA	89.75 ± 1.01 ^a^	87.05 ± 0.88 ^b^	90.16 ± 0.75 ^a^	89.46 ± 0.63 ^a^	89.74 ± 0.95 ^a^	89.86 ± 0.67 ^a^	89.33 ± 0.53 ^a^	90.09 ± 1.22 ^a^	90.05 ± 0.98 ^a^	1.07
MUFA	55.75 ± 0.71 ^bc^	48.08 ± 0.45 ^b^	56.12 ± 0.51 ^bc^	58.30 ± 0.78 ^a^	55.46 ± 0.27 ^bcd^	55.08 ± 0.32 ^bc^	55.89 ± 0.49 ^bc^	57.67 ± 0.85 ^ab^	56.25 ± 0.43 ^ab^	5.29
PUFA	34.00 ± 0.25 ^b^	38.97 ± 0.33 ^a^	34.04 ± 0.38 ^b^	34.00 ± 0.55 ^b^	34.66 ± 0.43 ^b^	31.56 ± 0.29 ^c^	33.44 ± 0.61 ^bc^	32.42 ± 0.41 ^c^	33.8 ± 0.26 ^bc^	6.04

Duncan’s multiple range test was used for analysis, and different lowercase letters in the same column indicate significant differences (*p* < 0.05, *n* = 3). SFA: saturated fatty acid; UFA: unsaturated fatty acid; MUFA: monounsaturated fatty acid; PUFA: polyunsaturated fatty acid; ND: detection results below the detection limit.

## Data Availability

Data is contained within the article or Supplementary Material.

## Supplementary Materials

The following are available online at https://www.mdpi.com/article/10.3390/foods12132444/s1, Table S1: The phenotypic traits of Acer truncatum Bunge fruits from different locations (X ± SD); Table S2: The phenotypic traits of Acer truncatum Bunge seeds from different locations; Table S3: The subordinative function values of Acer truncatum Bunge phenotypic traits; Table S4: The mean subordinative function values of Acer truncatum Bunge protein; Table S5: The mean subordinative function values of Acer truncatum Bunge oil.

## Abbreviations

ATB	Acer truncatum Bunge
C16:0	palmitic acid
C16:1	hexadecenoic acid
C17:0	heptadecanoic acid
C18:0	stearic acid
C18:1	oleic acid
C18:2	linoleic acid
C18:3	linolenic acid
C20:0	arachidic acid
C20:1	cis-11-eicosanoic acid
C22:0	behenic acid
C22:1	erucic acid
C24:0	xylic acid
C24:1	nervonic acid
SFA	saturated fatty acid
UFA	unsaturated fatty acid
MUFA	monounsaturated fatty acid
PUFA	polyunsaturated fatty acid

## References

[1] Bai Y., Zhai Y., Ji C., Zhang T., Chen W., Shen X., Hong J. (2021). Environmental sustainability challenges of China’s edible vegetable oil industry: From farm to factory. Resour. Conserv. Recycl..

[2] Fang C., Beghin J.C. (2002). Urban demand for edible oils and fats in China: Evidence from household survey data. J. Comp. Econ..

[3] Liang Q., Wang W., Yuan F., Liu X., Li D., Yang K.Q. (2019). Characterization of yuanbaofeng (*Acer truncatum* Bunge) samaras: Oil, fatty acid, and phytosterol content. Ind. Crops Prod..

[4] Li L., Manning W.J., Tong L., Wang X. (2015). Chronic drought stress reduced but not protected Shantung maple (*Acer truncatum* Bunge) from adverse effects of ozone (O3) on growth and physiology in the suburb of Beijing. China. Environ. Pollut..

[5] Gu R.H., Morcol T., Liu B., Shi M.J., Kennelly E.J., Long C.L. (2019). GC–MS, UPLC-QTOF-MS, and bioactivity characterization of *Acer truncatum* seeds. Ind. Crops Prod..

[6] Qiao Q., Wang X., Ren H., An K., Feng Z., Cheng T., Sun Z. (2019). Oil Content and nervonic acid content of *Acer truncatum* seeds from 14 regions in China. Hortic. Plant J..

[7] (2011). NMCPRC: National Health Commission of the People’s Republic of China. http://www.nhc.gov.cn/sps/s7891/201103/cffd9def6007444ea271189c18063b54.shtml.

[8] Song X., Li H., Li C., Xu J., Hu D. (2016). Effects of VOCs from leaves of *Acer truncatum* Bunge and Cedrus deodara on human physiology and psychology. Urban For. Urban Green.

[9] Ang X., Chen H., Xiang J.Q., Wei F., Quek S.Y. (2019). Preparation and functionality of lipase-catalysed structured phospholipid—A review. Trends Food Sci. Technol..

[10] Chen H., Wei F., Dong X.Y., Xiang J.Q., Quek S.Y., Wang X. (2017). Lipidomics in food science. Curr. Opin. Food Sci..

[11] Dong X.Y., Zhong J., Wei F., Lv X., Wu L., Lei Y., Liao B.S., Quek S.Y., Chen H. (2015). Triacylglycerol composition profiling and comparison of high-oleic and normal peanut oils. J. Am. Oil Chem. Soc..

[12] Dai Y.J., Jiang G.Z., Liu W.B., Abasubong K.P., Zhang D.D., Li X.F., Chi C., Liu W.B. (2022). Evaluation of dietary linoleic acid on growth as well as hepatopancreatic index, lipid accumulation oxidative stress and inflammation in Chinese mitten crabs (*Eriocheir sinensis*). Aquac. Rep..

[13] Fousekis P. (2022). Price risk connectedness in the principal olive oil markets of the EU. J. Econ. Asymmetries.

[14] Hu W., Fitzgerald M., Topp B., Alam M., O’Hare T.J. (2022). Fatty acid diversity and interrelationships in macadamia nuts. Lwt.

[15] Araújo R.G., Rodriguez-Jasso R.M., Ruiz H.A., Govea-Salas M., Pintado M.E., Aguilar C.N. (2020). Process optimization of microwave-assisted extraction of bioactive molecules from avocado seeds. Ind. Crops Prod..

[16] Wang H., Pampati N., McCormick W.M., Bhattacharyya L. (2016). Protein nitrogen determination by kjeldahl digestion and ion chromatography. J. Pharm. Sci..

[17] Kamphorst S.H., Amaral Júnior A.T.D., Vergara-Diaz O., Gracia-Romero A., Fernandez-Gallego J.A., Chang-Espino M.C., Buchaillot M.L., Rezzouk F.Z., Lima V.J.D., Serret M.D. (2022). Heterosis and reciprocal effects for physiological and morphological traits of popcorn plants under different water conditions. Agric. Water Manag..

[18] Grivet D., Climent J., Zabal-Aguirre M., Neale D.B., Vendramin G.G., Gonzalez-Martinez S.C. (2013). Adaptive evolution of Mediterranean pines. Mol. Phylogenet. Evol..

[19] Zacharias M., Pampuch T., Heer K., Avanzi C., Wurth D.G., Trouillier M., Bog M., Wilmking M., Schnittler M. (2021). Population structure and the influence of microenvironment and genetic similarity on individual growth at Alaskan white spruce treelines. Sci. Total Environ..

[20] Xu J.J., Zhu Y.H., Wang G. (2021). Comprehensive evaluation and phenotypic diversity analysis of camellia meiocarpa ln Guizhou. J. Zhejiang For. Sci. Technol..

[21] Rodriguez-Romero J.J., Duran-Castaneda A.C., Cardenas-Castro A.P., Sanchez-Burgos J.A., Zamora-Gasga V.M., Sayago-Ayerdi S.G. (2022). What we know about protein gut metabolites: Implications and insights for human health and diseases. Food Chem. X.

[22] Yang R., Zhang L., Li P., Yu L., Mao J., Wang X., Zhang Q. (2018). A review of chemical composition and nutritional properties of minor vegetable oils in China. Trends Food Sci. Technol..

[23] Tang Y., Ren J., Liu C., Jiang J., Yang H., Li J. (2021). Genetic characteristics and QTL analysis of the soluble sugar content in ripe tomato fruits. Sci. Hortic..

[24] Vichaiya T., Faiyue B., Rotarayanont S., Uthaibutra J., Saengnil K. (2022). Exogenous trehalose alleviates chilling injury of ‘Kim Ju’ guava by modulating soluble sugar and energy metabolisms. Sci. Hortic..

[25] Chang P., Ma J., Xin H., Wang S., Chen Z., Hong X., Zhang B., Li L. (2022). Comparative Study of the Fatty Acid Composition of the *Acer truncatum* Bunge from Different Producing Areas. Forests.

[26] Krummel B., von Hanstein A.S., Plotz T., Lenzen S., Mehmeti I. (2022). Differential effects of saturated and unsaturated free fatty acids on ferroptosis in rat beta-cells. J. Nutr. Biochem..

[27] Wang X., Sun B., Wei L., Jian X., Shan K., He Q., Huang F., Ge X., Gao X., Feng N. (2022). Cholesterol and saturated fatty acids synergistically promote the malignant progression of prostate cancer. Neoplasia.

[28] Pei Z., Zhang L., Fang C., Yang J., Li J., Zhao Y., Wu Y. (2021). Assessment of dietary intakes of total fat and fatty acids for residents in China in 2015–2018. J. Food Compos. Anal..

[29] Szajewska H., Szajewski T. (2016). Saturated fat controversy: Importance of systematic reviews and meta-analyses. Crit. Rev. Food Sci. Nutr..

[30] Frigolet M.E., Gutierrez-Aguilar R. (2017). The role of the novel lipokine palmitoleic acid in health and disease. Adv. Nutr..

[31] Sacks F.M., Lichtenstein A.H., Wu J.H.Y., Appel L.J., Creager M.A., Kris-Etherton P.M., Miller M., Rimm E.B., Rudel L.L., Robinson J.G. (2017). Dietary fats and cardiovascular disease: A presidential advisory from the american heart association. Circulation.

[32] Astrup A., Magkos F., Bier D.M., Brenna J.T., de Oliveira Otto M.C., Hill J.O., King J.C., Mente A., Ordovas J.M., Volek J.S. (2020). Saturated fats and health: A reassessment and proposal for food-based recommendations: JACC state-of-the-art review. J. Am. Coll. Cardiol..

[33] Tan C.X. (2019). Virgin avocado oil: An emerging source of functional fruit oil. J. Funct. Foods.

[34] Hernández D., Fernández-Puratich H., Rebolledo-Leiva R., Tenreiro C., Gabriel D. (2019). Evaluation of sustainable manufacturing of pellets combining wastes from olive oil and forestry industries. Ind. Crops Prod..

[35] De Souza C.O., Valenzuela C.A., Baker E.J., Miles E.A., Rosa Neto J.C., Calder P.C. (2018). Palmitoleic acid has stronger anti-inflammatory potential in human endothelial cells compared to oleic and palmitic acids. Mol. Nutr. Food Res..

[36] Song W., Zhang K., Xue T., Han J., Peng F., Ding C., Lin F., Li J., Sze F.T.A., Gan J. (2022). Cognitive improvement effect of nervonic acid and essential fatty acids on rats ingesting *Acer truncatum* Bunge seed oil revealed by lipidomics approach. Food Funct..

[37] Riecan M., Paluchova V., Lopes M., Brejchova K., Kuda O. (2022). Branched and linear fatty acid esters of hydroxy fatty acids (FAHFA) relevant to human health. Pharmacol. Ther..

[38] Babin F., Sarda P., Limasset B., Descomps B., Rieu D., Mendy F., Crastes de Paulet A. (1993). Nervonic acid in red blood cell sphingomyelin in premature infants: An index of myelin maturation?. Lipids.

[39] Martínez M., Mougan I. (1998). Fatty acid composition of human brain phospholipids during normal development. J. Neurochem..

[40] Cook C., Barnett J., Coupland K., Sargent J. (1998). Effects of feeding Lunaria oil rich in nervonic and erucic acids on the fatty acid compositions of sphingomyelins from erythrocytes, liver, and brain of the quaking mouse mutant. Lipids.

[41] Violi F., Nocella C., Loffredo L., Carnevale R., Pignatelli P. (2022). Interventional study with vitamin E in cardiovascular disease and meta-analysis. Free Radic. Biol. Med..

[42] Burton G.W., Joyce A., Ingold K.U. (2022). Is Vitamin E the only lipid-soluble, chain-breaking antioxidant in human blood plasma and erythrocyte membranes?. Arch. Biochem. Biophys..

[43] Poli V., Aparna Y., Madduru R., Motireddy S.R. (2022). Protective effect of Vitamin C and E on enzymatic and antioxidant system in liver and kidney toxicity of Cadmium in rats. Appl. Food Res..

[44] Dun Q., Yao L., Deng Z., Li H., Li J., Fan Y., Zhang B. (2019). Effects of hot and cold-pressed processes on volatile compounds of peanut oil and corresponding analysis of characteristic flavor components. Lwt.

[45] Mu H., Wei C., Xu W., Gao W., Zhang W., Mai K. (2020). Effects of replacement of dietary fish oil by rapeseed oil on growth performance, anti-oxidative capacity and inflammatory response in large yellow croaker Larimichthys crocea. Aquac. Rep..

[46] Zainal Z., Khaza’ai H., Kutty Radhakrishnan A., Chang S.K. (2022). Therapeutic potential of palm oil Vitamin E-derived tocotrienols in inflammation and chronic diseases: Evidence from preclinical and clinical studies. Food Res. Int..

