# Supplementary material for: Research on the Differences in Phenotypic Traits and Nutritional Composition of Acer Truncatum Bunge Seeds from Various Regions
Source: Foods. 2023 Jun 21;12(13):2444. doi: 10.3390/foods12132444 (PMC10340834; doi:10.3390/foods12132444)
Supplement: Supplementary file 1 [file foods-12-02444-s001.zip › foods-2442432-supplementary.pdf]

Supplementary Materials

Research on the Differences in Phenotypic Traits and Nutritional Composition of Acer Truncatum Bunge Seeds from Various Regions

Xiaona Le [1,2], Wen Zhang [2], Guotao Sun [1,2], Jinshuan Fan [3] and Mingqiang Zhu [1,2,3,*]

1 College of Mechanical and Electronic Engineering, Northwest A&F University, Yangling 712100, China; lxn@nwafu.edu.cn (X.L.); guotao_sun@nwsuaf.edu.cn (G.S.)
2 Northwest Research Center of Rural Renewable Energy Exploitation and Utilization of M.O.A., Northwest A&F University, Yangling 712100, China; wen30511011@163.com
3 College of Forestry, Northwest A&F University, Yangling 712100, China; fanjinshuan@163.com
* Correspondence: zmqsx@nwsuaf.edu.cn; Fax: +86-29-87092269

Table S1

The phenotypic traits of Acer truncatum Bunge fruits from different locations (X±SD).

Origin	Long diameter (mm)	Transverse diameter (mm)	Longitudinal diameter (mm)	Growth mark length (mm)	Open-angle (°)	Weight per hundred kernels (g)	Kernel Yield (%)
AT1	27.79±3.15^{cde}	8.52±0.82^{d}	4.66±0.30^{a}	6.26±0.37^{a}	92.00±7.75^{b}	18.20±0.93^{a}	65.07±2.00^{bc}
AT2	22.96±2.75^{g}	6.49±0.60^{e}	2.59±1.37^{c}	4.04±1.53^{c}	115.00±12.01^{ab}	3.56±0.11^{e}	26.60±0.81^{e}
AT3	32.36±3.36^{a}	10.75±1.37^{a}	3.90±0.70^{ab}	5.96±0.51^{ab}	99.00±11.59^{b}	18.07±0.47^{ab}	66.84±0.67^{ab}
AT4	31.22±3.08^{ab}	9.52±1.00b^{c}	4.54±0.54^{a}	5.80±0.98^{ab}	105.00±5.20^{ab}	14.55±0.02^{c}	61.36±1.90^{cd}
AT5	26.28±4.33^{de}	8.87±1.52^{cd}	4.10±1.29^{ab}	5.77±1.13^{ab}	106.00±9.00^{ab}	16.72±0.42^{b}	67.97±2.16^{ab}
AT6	30.09±1.89^{ab}	9.48±0.43^{bc}	4.32±0.46^{ab}	5.09±0.64^{b}	128.00±2.00^{a}	18.16±1.34^{a}	69.89±1.31^{a}
AT7	28.69±2.01^{bcd}	9.92±0.65^{ab}	4.43±0.60^{ab}	5.58±0.66^{ab}	98.00±6.32^{b}	18.80±1.51^{a}	67.63±3.84^{ab}
AT8	24.89±1.75^{fg}	8.78±0.80^{cd}	4.05±0.57^{ab}	5.11±0.57^{b}	110.00±2.24^{ab}	14.24±0.24^{c}	61.74±2.24^{cd}
AT9	25.24±3.00^{efg}	8.22±0.69^{d}	3.64±0.80^{b}	5.40±1.01^{ab}	91.00±6.44^{b}	10.93±0.51^{d}	60.06±3.80^{d}
CV (%)	11.35	13.45	15.58	11.96	11.8	33.34	21.81

Duncan's multiple range test was used for analysis, and different lowercase letters in the same column indicate significant differences ($P < 0.05$, n=50). CV: coefficient variation

Table S2

The phenotypic traits of Acer truncatum Bunge seeds from different locations (mean±SD).

Origin	Long diameter (mm)	Transverse diameter (mm)	Longitudinal diameter (mm)	Weight per hundred kernels (g)	Kernel Yield (%)
AT1	10.36±0.23^{a}	7.45±0.35^{a}	4.15±0.07^{ab}	14.27±0.40^{a}	65.43±2.00^{d}
AT2	6.59±0.19^{e}	4.44±0.17^{e}	1.30±0.07^{e}	1.60±0.38^{g}	45.29±0.81^{e}
AT3	9.63±0.25^{ab}	6.74±0.20^{cd}	3.40±0.16^{cd}	12.53±0.15^{c}	70.89±0.67^{b}
AT4	9.41±0.176^{b}	6.72±0.14^{bc}	3.89±0.19^{bc}	11.63±0.23^{d}	69.55±1.90^{bc}
AT5	9.60±0.23^{ab}	6.97±0.20^{abc}	4.21±0.14^{ab}	11.96±0.14^{d}	65.69±2.16^{d}
AT6	8.80±0.19^{c}	6.46±0.12^{cd}	3.92±0.14^{abc}	14.12±0.16^{a}	68.37±1.31^{c}
AT7	10.03±0.32^{ab}	7.35±0.18^{ab}	4.37±0.22^{a}	13.27±0.46^{b}	65.54±3.84^{d}
AT8	8.34±0.35^{cd}	6.19±0.16^{d}	3.60±0.13^{cd}	10.29±0.09^{e}	74.50±2.24^{a}
AT9	7.95±0.22^{d}	6.46±0.12^{cd}	3.79±0.15^{bcd}	8.51±0.10^{f}	66.36±3.80^{d}
CV (%)	13.2	13.57	25.46	36.12	12.54

Duncan's multiple range test was used for analysis, and different lowercase letters in the same column indicate significant differences ($P < 0.05$, n=50). CV: coefficient variation

Table S3

The subordinative function values of Acer truncatum Bunge phenotypic traits.

	Subordinative function value	AT1	AT2	AT3	AT4	AT5	AT6	AT7	AT8	AT9
Fruits	Long diameter	0.51	0	1	0.88	0.35	0.76	0.61	0.21	0.24
	Transverse diameter	0.48	0	1	0.71	0.56	0.7	0.81	0.54	0.41
	Longitudinal diameter	1	0	0.63	0.94	0.73	0.84	0.89	0.71	0.51
	Weight per hundred kernels	0.96	0	0.95	0.72	0.86	0.96	1	0.7	0.48
	Kernel Yield	0.89	0	0.93	0.8	0.96	1	0.95	0.81	0.77
Seeds	Long diameter	1	0	0.81	0.75	0.8	0.59	0.91	0.46	0.36
	Transverse diameter	1	0	0.76	0.76	0.84	0.67	0.97	0.58	0.67
	Longitudinal diameter	0.93	0	0.68	0.84	0.95	0.85	1	0.75	0.81
	Weight per hundred kernels	1	0	0.86	0.79	0.82	0.99	0.92	0.69	0.55
	Kernel Yield	0.69	0	0.88	0.83	0.7	0.79	0.69	1	0.72
mean		0.84	0	0.85	0.8	0.76	0.81	0.87	0.64	0.55
Comprehensive ranking		3	9	2	5	6	4	1	7	8

Table S4

The mean subordinative function values of Acer truncatum Bunge protein.

Subordinative function value	AT1	AT2	AT3	AT4	AT5	AT6	AT7	AT8	AT9
The mean value of phenotypic traits of seed fruits	0.84	0.00	0.85	0.80	0.76	0.81	0.87	0.64	0.55
The content of protein	0.23	0.00	0.97	1.00	0.59	0.63	0.93	0.12	0.63
Mean	0.54	0.00	0.92	0.91	0.67	0.72	0.90	0.38	0.59
Comprehensive ranking	7	9	1	2	5	4	3	8	6

Table S5

The mean subordinative function values of Acer truncatum Bunge oil.

Subordinative function value	AT1	AT2	AT3	AT4	AT5	AT6	AT7	AT8	AT9
The mean value of phenotypic traits of seed fruits	0.84	0.00	0.85	0.80	0.76	0.81	0.87	0.64	0.55
The content of oil	0.81	0.00	1.00	0.89	0.83	0.78	0.77	0.73	0.80
The content of UFA	0.87	0.00	1.00	0.78	0.87	0.90	0.73	0.8	0.97
The content of C24:1	0.52	0.08	0.34	0.44	0.00	0.95	0.71	1.00	0.63
Mean	0.76	0.02	0.80	0.73	0.61	0.86	0.77	0.84	0.74
Comprehensive ranking	5	9	3	7	8	1	4	2	6
